# Supplementary material for: Chromoplast plastoglobules recruit the carotenoid biosynthetic pathway and contribute to carotenoid accumulation during tomato fruit maturation
Source: PLoS One. 2022 Dec 6;17(12):e0277774. doi: 10.1371/journal.pone.0277774 (PMC9725166; doi:10.1371/journal.pone.0277774)
Supplement: S1 Fig — (A) FBN heatmap and (B) ABC1K-like kinase heatmap were generated from peptide counts obtained from PG isolated from chloroplast and chromoplast, respectively. (PDF) [file pone.0277774.s001.pdf]

A

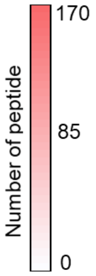

Accession No	Protein Name	PG chloroplast	PG chromoplast
Solyc02g081170.2.1	FBN1a/b		
Solyc08g076480.2.1	FBN2		
Solyc09g090330.2.1	FBN4		
Solyc10g080490.1.1	FBN7a		
Solyc03g062790.2.1	FBN7b		
Solyc08g068590.2.1	FBN8		

B

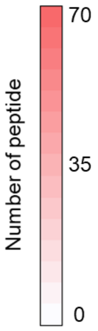

Accession No	Protein Name	PG chloroplast	PG chromoplast
Solyc08g074560.2.1	ABC1K1		
Solyc04g083010.2.1	ABC1K3		
Solyc04g072230.2.1	ABC1K5		
Solyc09g091580.2.1	ABC1K6		
Solyc07g045420.2.1	ABC1K7		
Solyc03g095620.2.1	ABC1K9		

S1 Fig
