# Supplementary figures and images for: Chromoplast plastoglobules recruit the carotenoid biosynthetic pathway and contribute to carotenoid accumulation during tomato fruit maturation
Source: PLoS One. 2022 Dec 6;17(12):e0277774. doi: 10.1371/journal.pone.0277774 (PMC9725166; doi:10.1371/journal.pone.0277774)

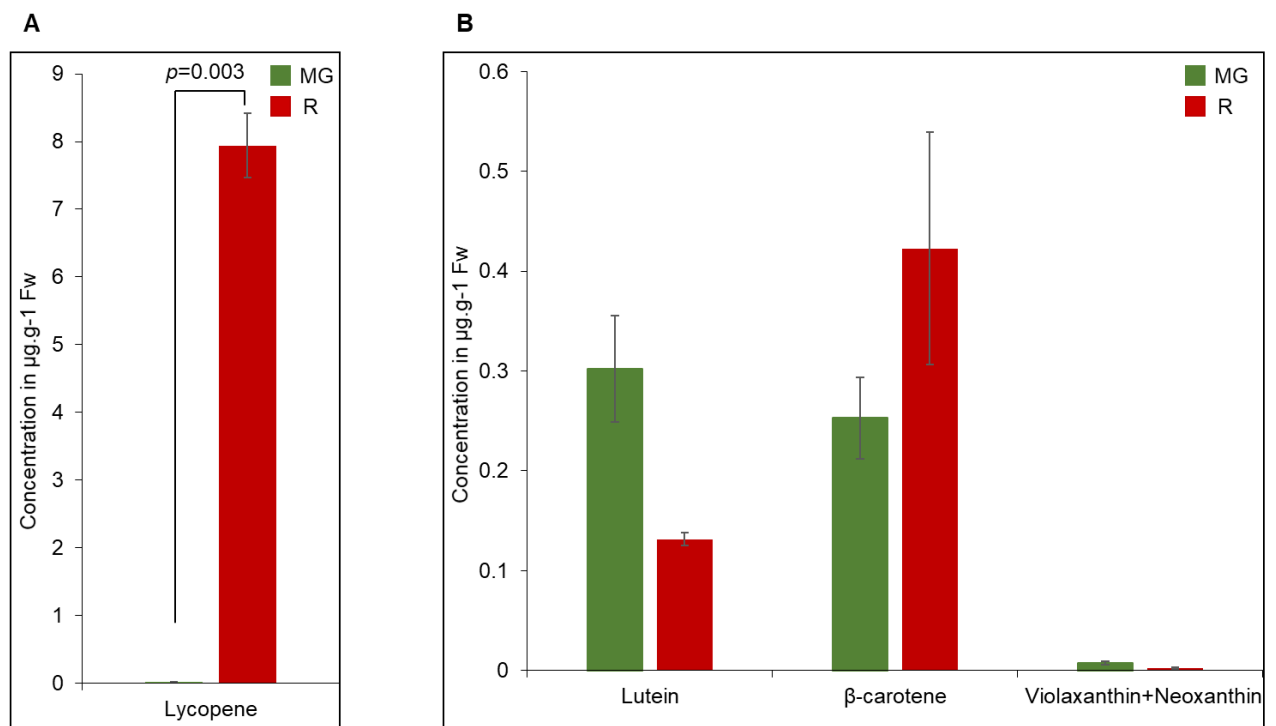

**Fig S2**

Supplement: S2 Fig — (A) Total carotenoids were extracted from mature green (MG) and red (R) tomato fruit and lycopene was quantified. (B) Quantification of lutein, β-carotene, and violaxanthin/neoxanthin. All values in the figure are the mean of 3 biological replicates (n = 3). Statistical differences were assessed with student’s t test and p values are indicated. (PDF) [file pone.0277774.s002.pdf]

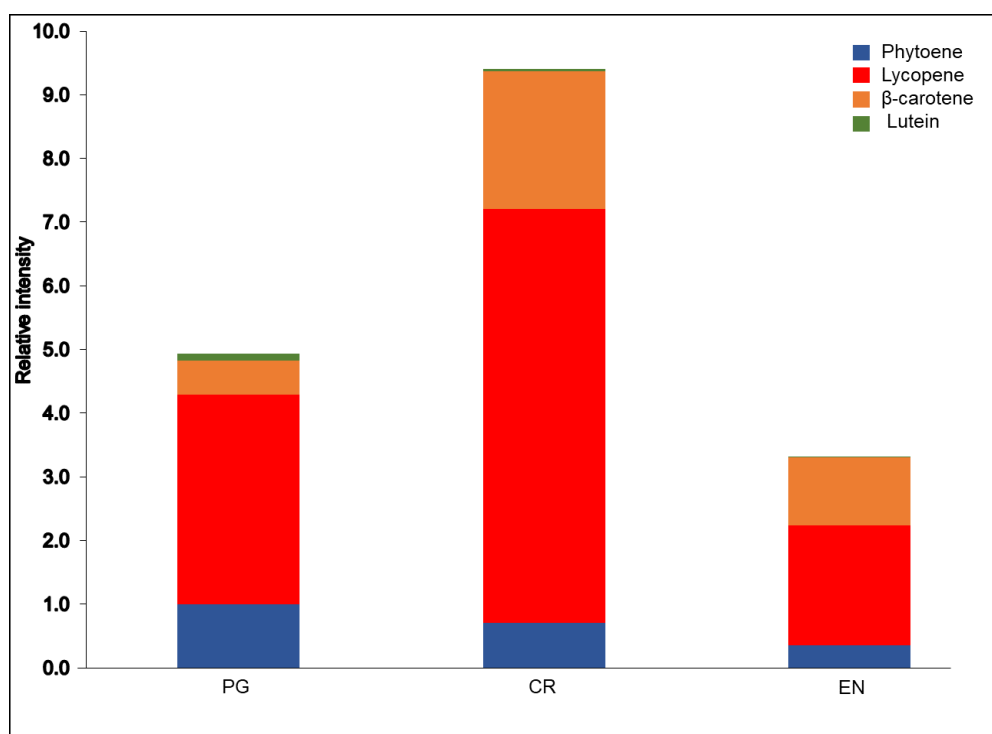

**Fig S3**

Supplement: S3 Fig — The total carotenoids were extracted from equal volumes of mature red (R) tomato fruit PG (plastoglobules); CR (carotenoid crystals) fractions; EN (envelope). The isolated lycopene, phytoene, β-carotene, and lutein were quantified. Values are the mean of 3 biological replicates (n = 3). (PDF) [file pone.0277774.s003.pdf]

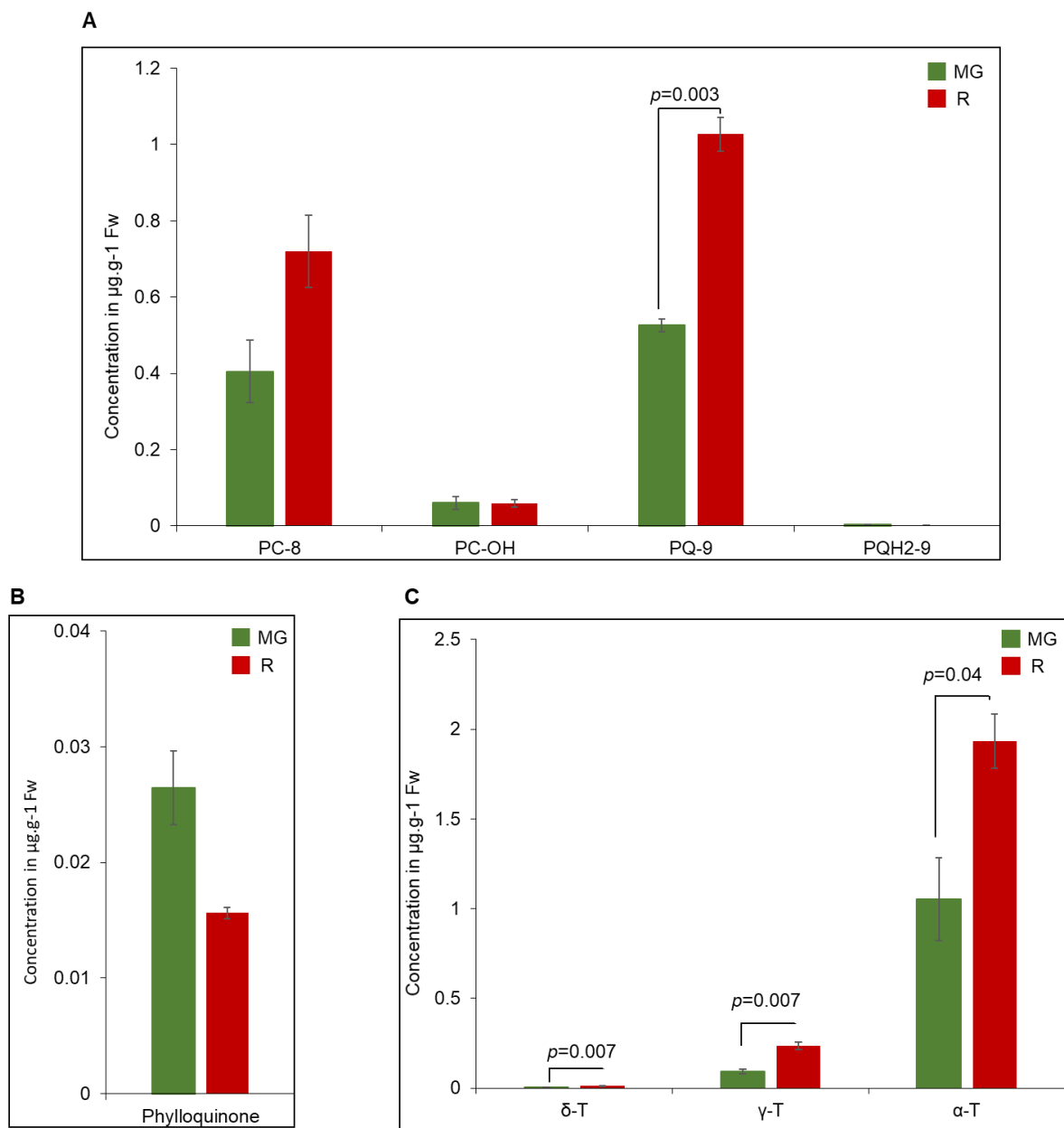

**Fig S4**

Supplement: S4 Fig — (A) The total prenyl quinones were extracted from mature green (MG) and red (R) tomato fruit, PC-8, plastochromanol; PC-OH, hydroxy-plastochromanol; PQ-9, plastoquinone; and PQH2-9, plastoquinol were quantified (B) Quantification of phylloquinone. (C) Quantification of tocopherols. All values in the figure are the mean of 3 biological replicates (n = 3). Statistical differences were assessed with student’s t test and p values are indicated. (PDF) [file pone.0277774.s004.pdf]
