# Supplementary material for: Chromoplast plastoglobules recruit the carotenoid biosynthetic pathway and contribute to carotenoid accumulation during tomato fruit maturation
Source: PLoS One. 2022 Dec 6;17(12):e0277774. doi: 10.1371/journal.pone.0277774 (PMC9725166; doi:10.1371/journal.pone.0277774)

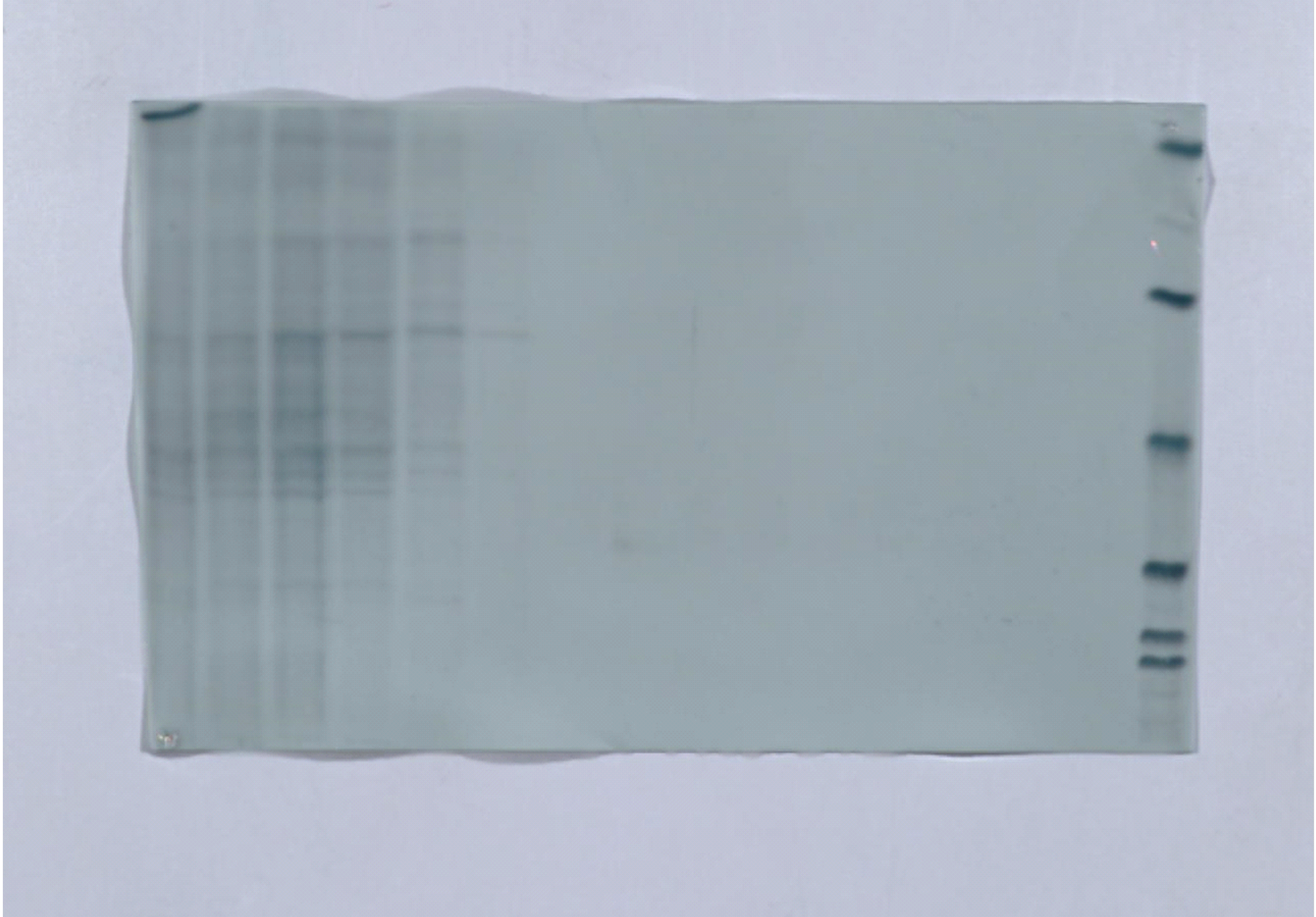

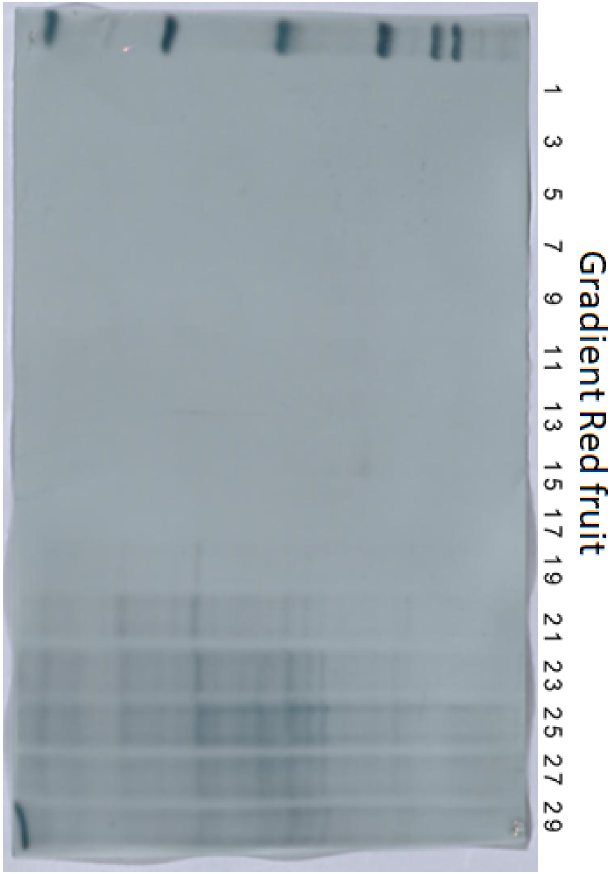

1 3 5 7 9 11 13 15 17 19 21 23 25 27 29
Gradient Red fruit

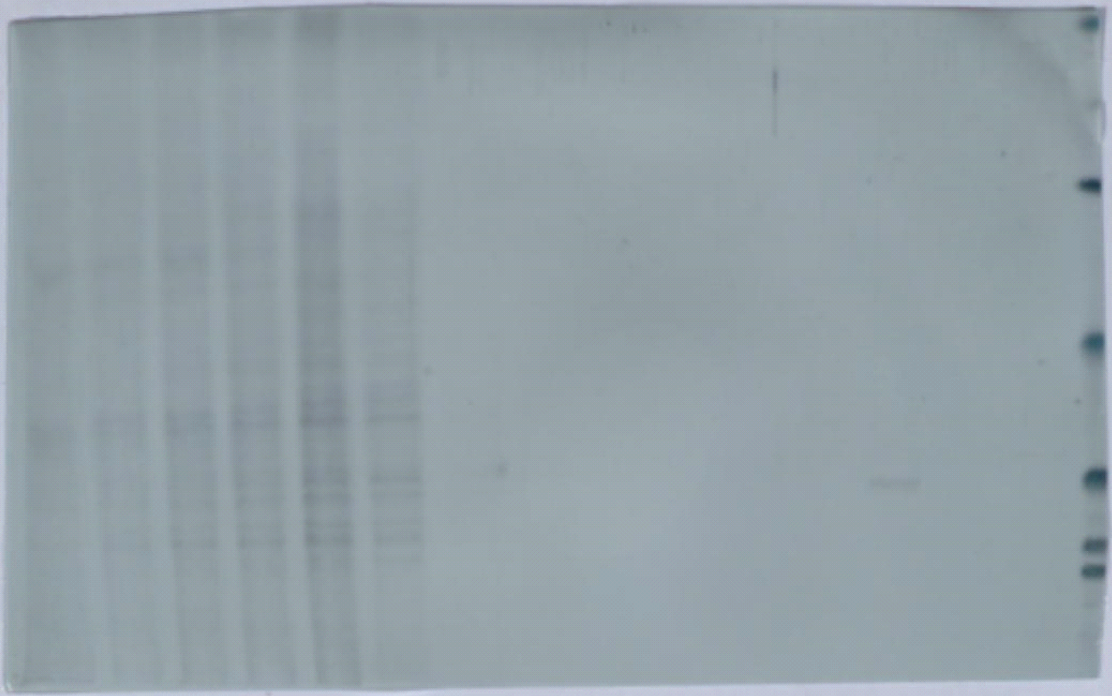

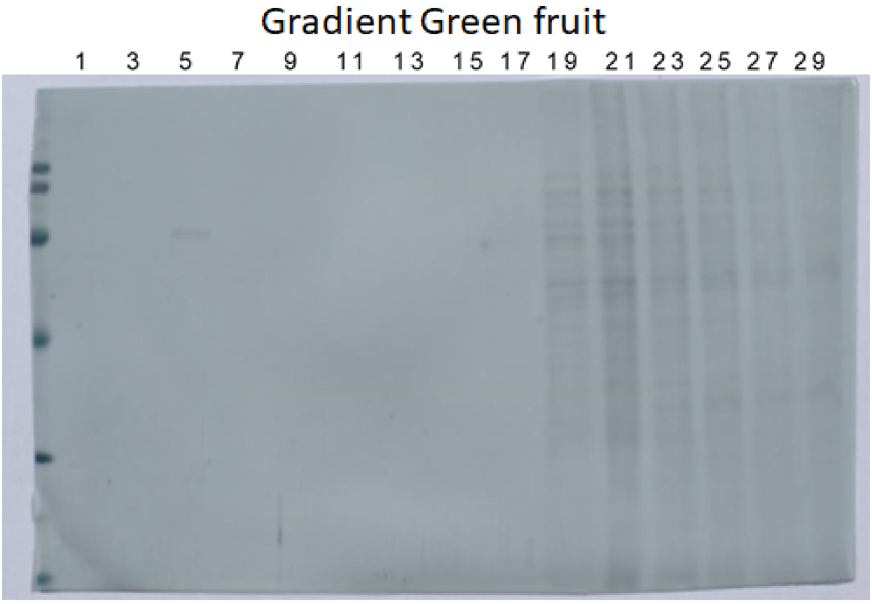
Gradient Green fruit
1 3 5 7 9 11 13 15 17 19 21 23 25 27 29

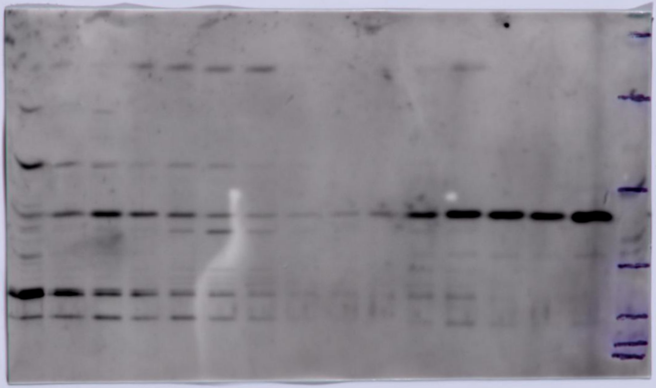

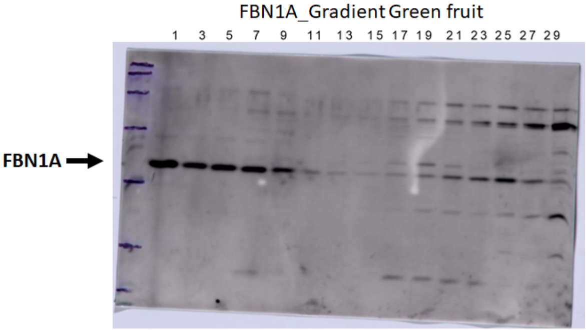
FBN1A_Gradient Green fruit
1 3 5 7 9 11 13 15 17 19 21 23 25 27 29
FBN1A

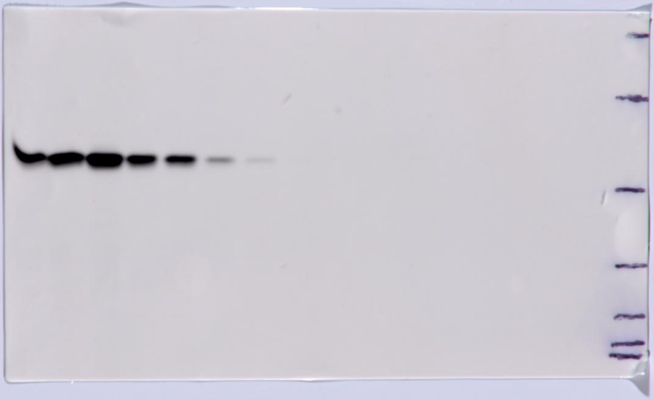

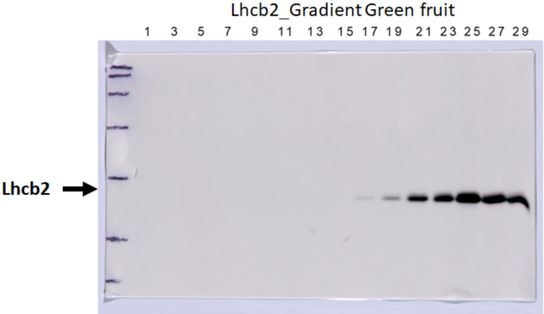
Lhcb2_Gradient Green fruit
1 3 5 7 9 11 13 15 17 19 21 23 25 27 29
Lhcb2

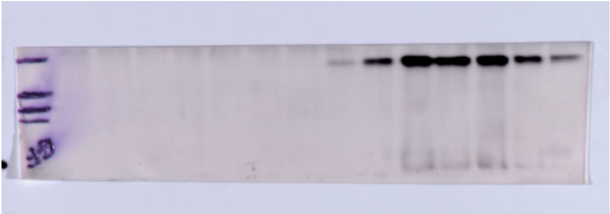

TOC75_Gradient Green fruit

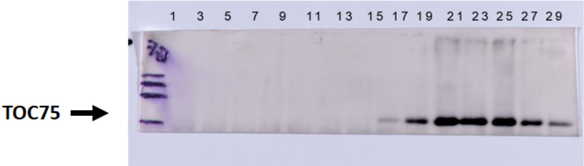

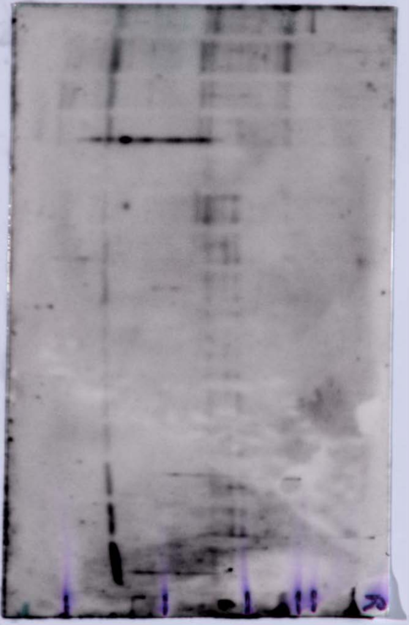

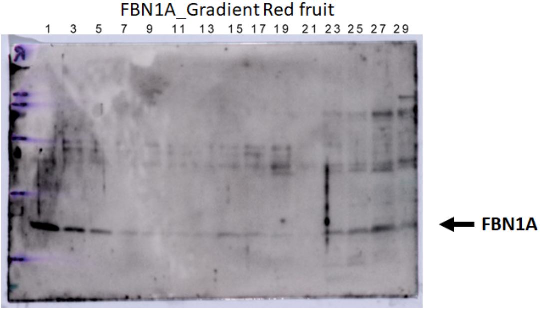
FBN1A_Gradient Red fruit
1 3 5 7 9 11 13 15 17 19 21 23 25 27 29
FBN1A

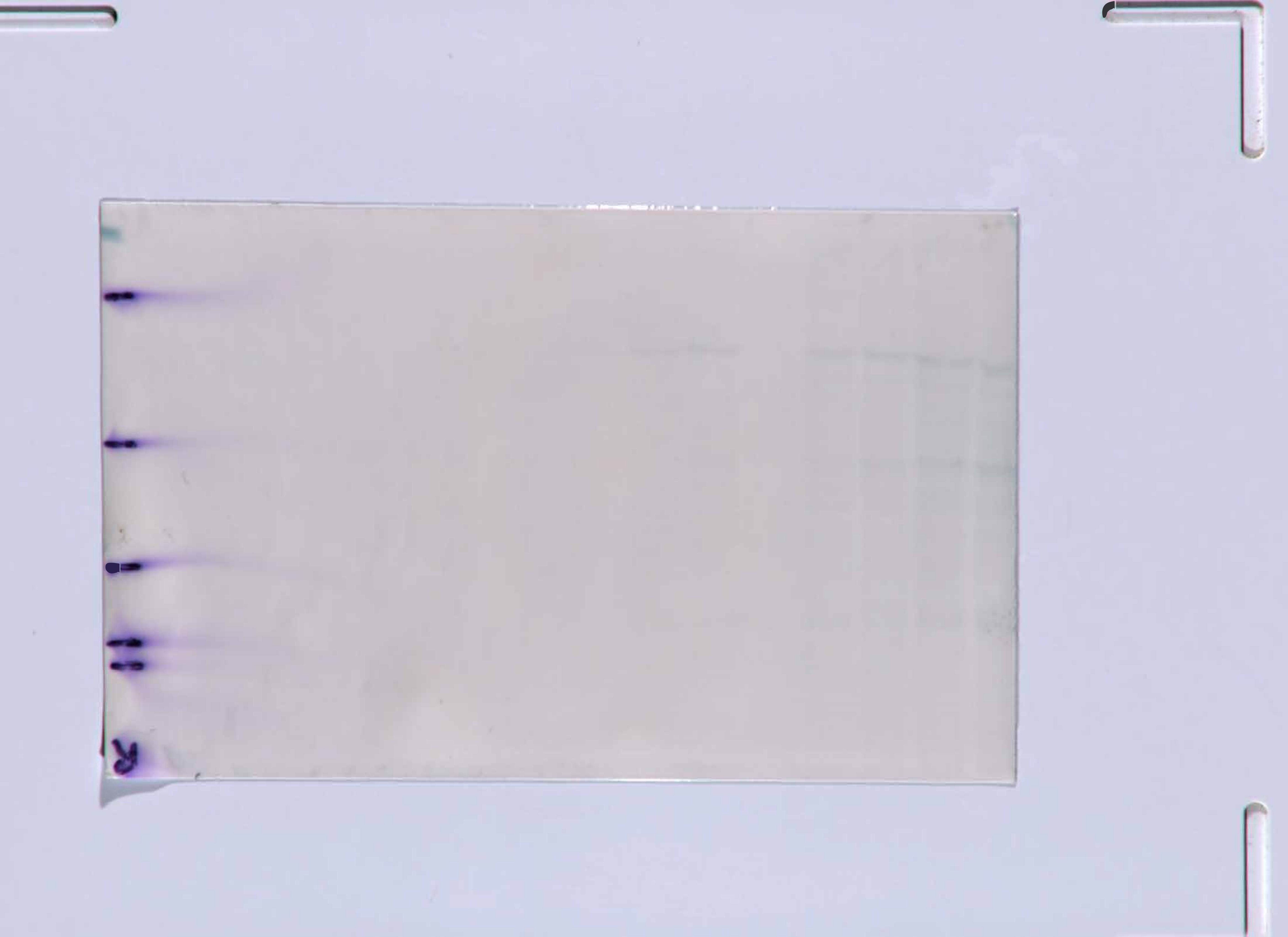

Lhcb2_Gradient Red fruit

1 3 5 7 9 11 13 15 17 19 21 23 25 27 29

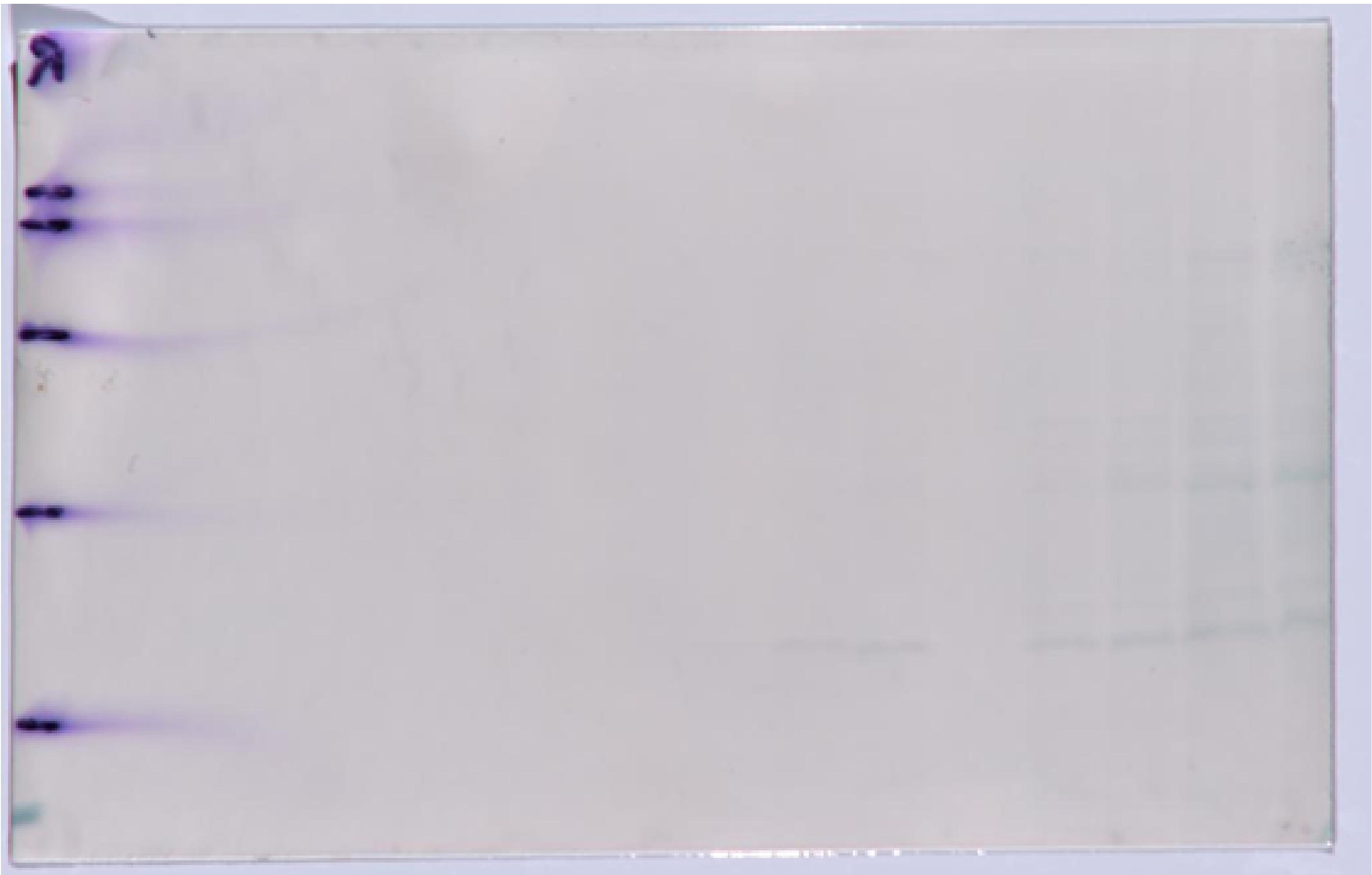

Lhcb2_Gradient Red fruit

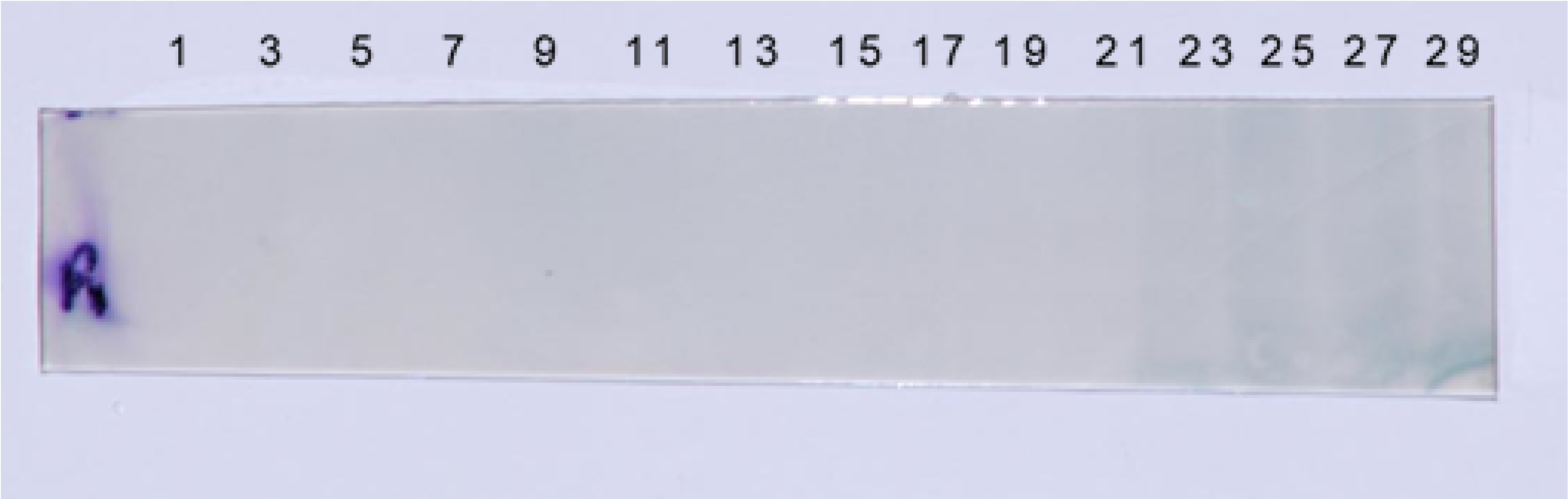

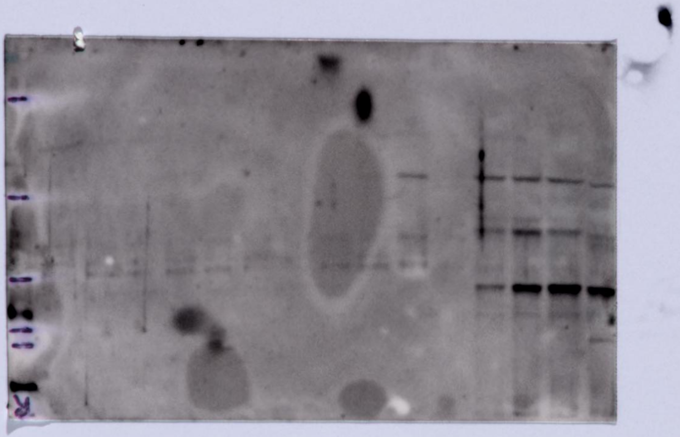

TOC75_Gradient Red fruit

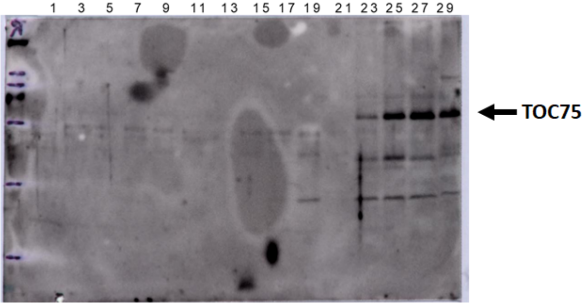

Supplement: S1 Raw images — (PDF) [file pone.0277774.s006.pdf]
